# A thermosensitive mutation alters the effects of lacosamide on slow inactivation in neuronal voltage-gated sodium channels, Na_V_1.2

**DOI:** 10.3389/fphar.2013.00121

**Published:** 2013-09-20

**Authors:** Mena Abdelsayed, Stanislav Sokolov, Peter C. Ruben

**Affiliations:** Department of Biomedical Physiology and Kinesiology, Simon Fraser UniversityBurnaby, BC, Canada

**Keywords:** voltage-gated sodium channels, slow inactivation, lacosamide, GEFS+, thermosensitive, WT-β1 (wild-type), C121W-β1

## Abstract

Epilepsy is a disorder characterized by seizures and convulsions. The basis of epilepsy is an increase in neuronal excitability that, in some cases, may be caused by functional defects in neuronal voltage gated sodium channels (Na_Vs_). The C121W mutation of the β1 subunit, in particular, gives rise to the thermosensitive generalized epilepsy with febrile seizures plus (GEFS+) phenotype. Lacosamide is used to treat epileptic seizures and is distinct from other anti-seizure drugs by targeting Na_V_ slow-inactivation. We studied the effects of a physiologically relevant concentration of lacosamide on the biophysical properties of Na_V_1.2 channels associated with either WT-β1 or the mutant C121W-β1 subunit. Biophysical parameters were measured at both normal (22°C) and elevated (34°C) temperatures to elicit the differential temperature-sensitivity of C121W. Lacosamide was more effective in Na_V_1.2 associated with the WT-β1 than with C121W-β1 at either temperature. There is also a more potent effect by lacosamide on slow inactivation at elevated temperatures. Our data suggest a modulatory role is imparted by the β1 subunit in the interaction between the drug and the channel.

## Introduction

Epilepsy is a neurological disorder in which neuronal hyperexcitability causes convulsions and seizures. This disorder may be elicited via different mechanisms, some of which have genetic origins. There are over 150 described epileptogenic mutations in the Na_V_1.1 isoform of the voltage-gated sodium channel (Na_V_), which is expressed in the central nervous system (Clare et al., 2000; Meisler and Kearney, 2005; Mantegazza et al., 2010). Eight epileptogenic mutations are associated with Na_V_1.2 (Meisler and Kearney, 2005). Several mutations/deletions in the auxiliary β1 subunit give rise to generalized epilepsy with febrile seizures plus (GEFS+) (Wallace et al., 1998; Audenaert et al., 2003; Xu et al., 2007). The C121W-β1 was the first of such mutations to be described (Wallace et al., 1998; Wimmer et al., 2010), and causes channel hyperexcitabilty at elevated temperatures. It was later demonstrated that the kinetics of recovery from the fast-inactivated state is enhanced at elevated temperatures (34°C) when the C121W-β1 mutation is co-expressed with Na_V_1.2 (Egri et al., 2012). The WT-β1 subunit thus appears to have a thermoprotective function that is lost in C121W-β1.

Two classes of anticonvulsants act on different time-scales of inactivation in neuronal Na_V_s. The classic anticonvulsants, including phenytoin, carbamazepine, and lamotrigine, stabilize the fast-inactivated state (Ragsdale et al., 1996; Ragsdale and Avoli, 1998; Lees and Shipton, 2009; Karoly et al., 2010). In fast inactivation, the IFMT motif in the cytoplasmic linker between domains III and IV occludes the pore of the channel in the millisecond time scale via thermodynamically favorable interactions with the inner vestibule of the channel (Ragsdale and Avoli, 1998; Clare et al., 2000; Denac et al., 2000; Yu et al., 2005; Catterall et al., 2012). Classic anticonvulsants bind within the inner vestibule of the channel forming interactions with the IFMT and fast-inactivation binding sites of the IFMT motif (Ragsdale and Avoli, 1998). A novel class of anticonvulsants, including lacosamide, act by a different mechanism to stabilize the slow-inactivated state of Na_*V*_s (Errington et al., 2008; Sheets et al., 2008; Lees and Shipton, 2009). Slow inactivation occurs over a hundred-milliseconds to seconds time scale through a combination of conformational rearrangements in the pore region and long distance interactions with other channel structures (O'reilly et al., 1999; Ong et al., 2000; McCollum et al., 2003; Zhang et al., 2003).

We tested the effects of lacosamide on Na_V_1.2 co-expressed with both WT-β1 and C121W-β1 subunits, at normal (22°C) and elevated temperatures (34°C) to mimic the temperature sensitivity of GEFS+. We find the efficacy of lacosamide is lost with the C121W-β1 mutation. This suggests the β1 subunit modulates the interaction between lacosamide and the Na_V_1.2 α-subunit.

## Methods

### Cell culture

Chinese hamster ovary (CHO) cells stably expressing the rat Na_V_1.2 channel (a gift from W.A. Catterall) were grown in filter sterilized DMEM/F12 (Invitrogen, Carlsbad, CA, USA) with glutamine, supplemented with 2 g/L NaCHO3, 100 units/ml penicillin, 0.01 mg/ml streptomycin, 50 mg/ml G418 at pH 7.4, 5% FBS and maintained in a humidified environment at 37°C with 5% CO_2_. Cells were plated on glass cover slips at a density conducive to single cell isolation.

### Transfection

The stably expressing Na_V_1.2 cell line was transiently transfected with either WT-β1 rat subunit (pBK/CMV vector) or the mutant rat ortholog of the C121W-β1 (pBK/CMV), and enhanced green fluorescent protein (eGFP) according to the PolyFect transfection protocol (Invitrogen).

### Electrophysiology

Whole-cell recordings were performed in an extracellular solution containing (in mM): 140 NaCl, 4 KCl, 2 CaCl_2_, 1 MgCl_2_, 10 HEPES. Solutions were adjusted to pH 7.4 with CsOH. Patch pipettes were fabricated using a P-97 puller and borosilicate glass (Sutter Instruments, CA, USA) using a Model P-1000 Puller (Sutter Instruments), dipped in dental wax to reduce capacitance, and thermally polished to a resistance of 1.0–1.5 MΩ. Pipette (intracellular) solution was composed of the following (in mM): 120 CsF, 20 CsCl, 10NaCl, and 10 HEPES adjusted to pH 7.4 with CsOH. Dimethyl sulfoxide (DMSO) was used as solvent for lacosamide and diluted in bath solution at < 1:1000 (by volume) from stock to obtain a 100 μM final drug concentration. Stock lacosamide solution was pre-aliquoted in extracellular solution. All recordings were made using an EPC-9 patch-clamp amplifier (HEKA Elektronik, Lambrecht, Germany) digitized at 200 kHz via an ITC-16 interface (Instrutech, Great Neck, NY, USA). Voltage clamping and data acquisition were controlled using PatchMaster/FitMaster software (HEKA Elektronik, Lambrecht, Germany) running on an Apple iMac. Current was low-pass-filtered at 5 kHz. Leak subtraction was performed automatically by software using a P/4 procedure following the test pulse. Bath solution was maintained at 22°C ± 0.2°C or 34°C ± 0.2°C using a Peltier device controlled by an HCC-100A temperature controller (Dagan, Minneapolis, MN, USA). Experiments were not performed at higher temperatures because of inherent instability of the temperature controller and cell membranes beyond 34°C.

Gigaohm seals were allowed to stabilize in the on-cell configuration for 1 min prior to establishing the whole-cell configuration. We ensured that cells used for recordings had *R*-series <3.5 MΩ for recordings. Series resistance compensation up to 80% was used when necessary. All data were acquired >5 min after attaining the whole-cell configuration, and cells were allowed to incubate 5 min after drug application prior to data collection. Before each protocol, the membrane potential was hyperpolarized to −110 mV to insure complete removal of both fast-inactivation and slow-inactivation.

### Analysis

Analysis and graphing were done using FitMaster software (HEKA Elektronik, Lambrecht, Germany) and Igor Pro (Wavemetrics, Lake Oswego, OR, USA) with statistical information derived using InStat (Graphpad Software Inc., San Diego, CA, USA). All data acquisition and analysis programs were run on an Apple iMac (Apple Computer, Cupertino, CA). Exponential or Boltzmann fits were performed for individual data sets to obtain means for time constants, apparent valence (z), and midpoints of voltage-dependence (*V*_1/2_). Statistical significance was accepted at *p* < 0.05 using Student's unpaired “t” tests with two-tailed *p*-values. All values reported are given as means ± standard error of means.

### Voltage protocols

#### Activation

To determine the voltage dependence of activation, we measured the peak current amplitude at test pulse potentials ranging from −80 mV to +60 mV in increments of +10 mV for 20 ms. Channel conductance (G) was calculated from peak *I*_Na_.

$$G_{\text{Na}}=I_{\text{Na}}/\text{​}V-E_{\text{Na}}$$

where *G*_Na_ is conductance, *I*_Na_ is peak sodium current in response to the command potential *V*, and *E*_Na_ is the Nernst equilibrium potential. Calculated values for conductance were fit with the Boltzmann equation:

$$G/\text{​}G_{\text{max}}=\text{1}/\text{​}\left(\text{1}+\text{exp​}\left[-ze_{0}\left[V_{m}-V_{\text{1/2}}\right]\text{​}/kT\right]\right)$$

where *G*/*G*_max_ is normalized conductance amplitude, *V*_*m*_ is the command potential, *z* is the apparent valence, *e*_0_ is the elementary charge, *V*_1/2_ is the midpoint voltage, *k* is the Boltzmann constant, and *T* is temperature in °*K*.

#### Steady-state fast inactivation

The voltage-dependence of fast inactivation was measured by preconditioning the channels to a hyperpolarizing potential of −130 mV and then eliciting prepulse potentials that ranged from −130 to +10 mV in increments of 10 mV for either 20 ms or 500 ms, followed by a 10 ms test pulse during which the voltage was stepped to 0 mV. Normalized current amplitude as a function of voltage was fit using the Boltzmann equation:

$$I\text{​}/\text{​}I_{\text{max}}=\text{1​}/\text{​}(\text{1}+\text{exp}\left(-ze_{0}\left(V_{M}-V_{\text{1/2}}\right)\text{​}/kT\right)$$

where *I*_max_ is the maximum test pulse current amplitude.

#### Open-state fast inactivation

Time constants for open state fast-inactivation were derived by fitting a single exponential function to the decay of current from the peak to the end of the depolarizing stimulus.

#### Recovery from fast inactivation

Channels were fast-inactivated during a 20 ms or 500 ms depolarizing step to 0 mV, and recovery was measured during a 19 ms test pulse to 0 mV following a −110 mV recovery pulse for durations between 0 and 1.024 s. Time constants of fast inactivation recovery showed two components and were fit using a double exponential equation:

$$I=I_{\text{ss}}+\alpha_{1}\text{exp​}\left(-t/\tau_{1}\right)+\alpha_{2}\text{exp}\left(-t/\tau_{2}\right)$$

where I is current amplitude, *I*_ss_ is the plateau amplitude, α_1_ and α_2_ are the amplitudes at time 0 for time constants τ_1_ and τ_2_, and *t* is time.

#### Steady-state slow inactivation

The voltage-dependence of slow inactivation was measured by preconditioning the cells −130 mV for 30 s. We then delivered prepulse potentials from −130 mV to +10 mV, alternating with every sweep, in increments of 20 mV. After the prepulse, a 100 ms pulse at −130 mV was applied to recover channels from fast inactivation prior to a 20 ms test pulse at 0 mV. Peak current amplitude during the test pulse was normalized to that measured following the most negative prepulse, and plotted as a function of prepulse potential. Steady-state slow inactivation curves were fit with the following modified Boltzmann equation that takes into account changes in the steady-state probability of slow inactivation:

$$I\text{​}/\text{​}I_{\text{max}}=\left(I_{1}-I_{2}\right)\text{​​}/\text{​}(\text{1}+\text{exp​}\left(-ze_{0}\left(V_{\text{m}}-V_{\text{1/2}}\right)\text{​}/kT\right)+I_{2}$$

where *I*/*I*_max_ is the maximum probability, *I*_1_ and *I*_2_ are maximum and minimum values in the fit, respectively, z is apparent valence, *e*_0_ is the elementary charge, *V*_m_ is the prepulse potential, *V*_1/2_ is the midpoint voltage of the steady-state slow inactivation curve, *k* is the Boltzmann constant, and *T* is the absolute temperature.

#### Onset of slow inactivation

A 5 ms test pulse to 0 mV measured the rate of slow-inactivation onset following a two-pulse protocol consisting of 0–64 s durations at the conditioning voltage (0 mV) and a 100 ms or 2 s hyperpolarizing pulse to −110 mV (“recovery pulse”). Time constants of slow-inactivation onset as a function of time were fit using a double exponential equation.

#### Recovery from slow inactivation

Slow inactivation was induced with either a 500 ms or 8 s depolarizing pulse to 0 mV, after which the membrane was hyperpolarized to −110 mV for durations of 0.02–60 s and peak current was tested with a 5 ms pulse to 0 mV. Time constants of slow inactivation recovery, plotted as a function of time, were fit with a double exponential equation.

## Results

### Activation

**Figure 1:**
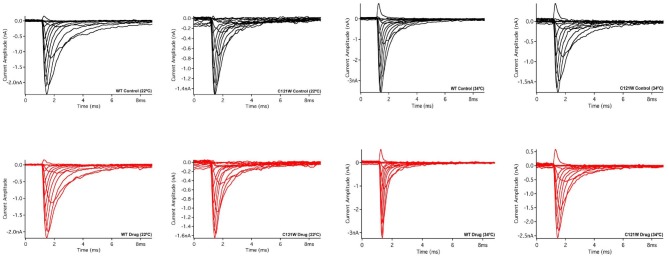
**Macroscopic currents**. Current amplitude plotted verses time duration (ms). These traces were elicited by a set of alternating test pulses that range from −80 mV to +60 mV in increments of +10 mV.

Lacosamide (100 μM) caused no significant effect on the voltage dependence of activation. There were no significant shifts in the midpoint (*V*_1/2_) or apparent valence (*z*) at either elevated or normal temperatures in the WT-β1 and the C121W-β1 subunits (Table 1). Current traces from the four experimental conditions are shown in Figure 1.

**Table 1:** **Activation parameters**.

	***V*_1/2_ (mV)**	***z* (slope)**	***n***
**22°C**			
WT-β1 Control	−19.4 ± 0.7	4.1 ± 0.2	12
WT-β1 Drug	−19.2 ± 2.0	3.7 ± 0.2	6
C121W-β1 Control	−16.0 ± 1.0	3.3 ± 0.1	10
C121W-β1 Drug	−16.8 ± 1.2	3.5 ± 0.4	6
**34°C**			
WT-β1 Control	−19.7 ± 0.4	6.1 ± 0.2	17
WT-β1 Drug	−21.1 ± 0.5	6.6 ± 0.3	11
C121W-β1 Control	−18.8 ± 0.3	7.1 ± 0.2	19
C121W-β1 Drug	−21.5 ± 0.6	6.3 ± 0.4	9

### Fast inactivation

**Figure 2:**
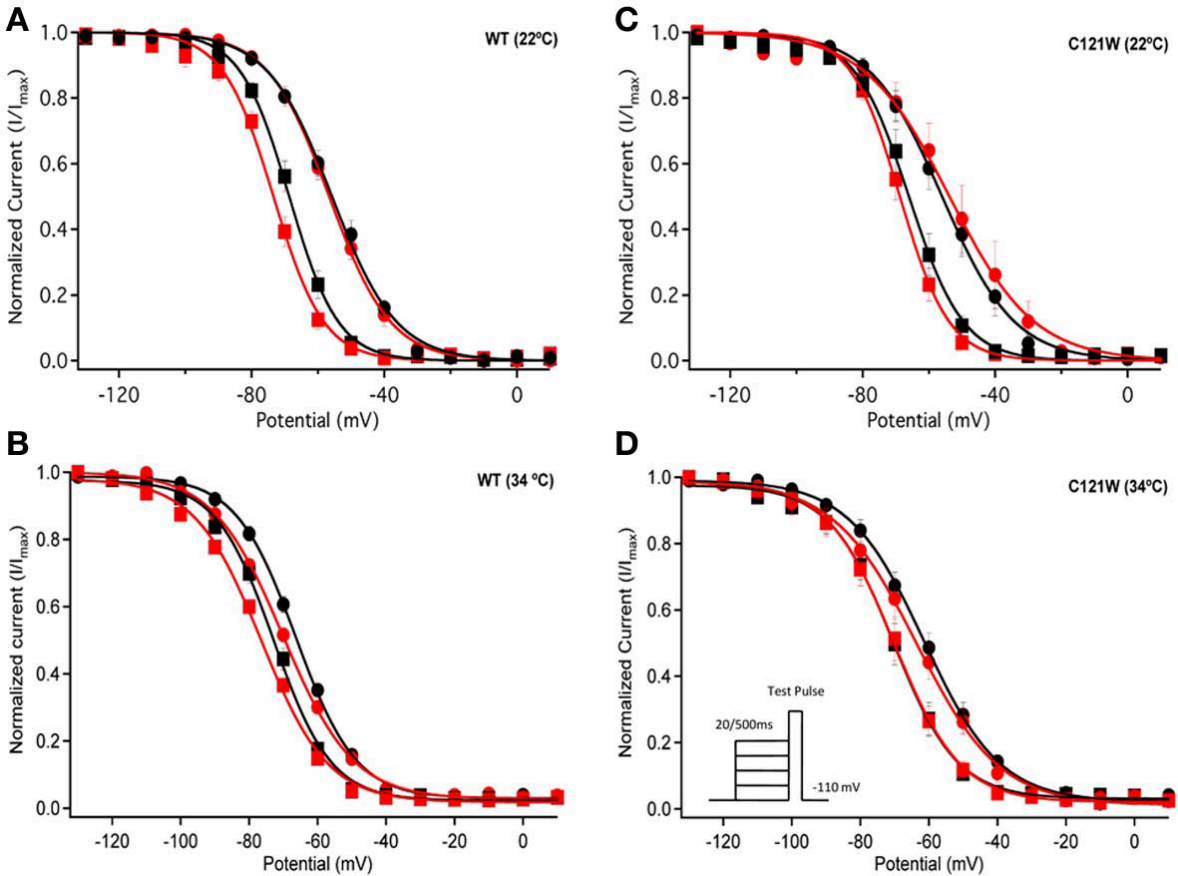
**Steady-state fast inactivation**. Normalized current is plotted against a range of prepulse potentials (mV). Steady-state fast inactivation was elicited by both 20 ms prepulse durations (filled circles) and 500 ms prepulse durations (filled squares). For both WT at 22°C and 34°C **(A,B)** and C121W at 22°C and 34°C **(C,D)**. The means of control (shown in black) were compared to 100 μM Lacosamide (shown in red). In panel **(D)**, an inset shows the pulse protocol used to measure steady-state fast inactivation.

Lacosamide did not cause significant shifts in the kinetics of open state fast inactivation parameters in Na_V_1.2 co-expressed with either WT-β1 or C121W-β1 (Table 2). We examined steady-state fast inactivation with both 20 ms and 500 ms prepulse duration. There were no statistically significant shifts in the midpoint or apparent valence between control and 100 μM lacosamide for WT-β1 at 22°C with prepulse durations of 20 ms (Figure 2A, Table 3). At 34°C there was a significant shift in the midpoint value (Figure 2B, Table 3). However, there was no shift in the apparent valence with drug perfusion. Stabilization of steady-state fast inactivation was observed with 500 ms prepulse depolarizations. At both 22°C and 34°C, there were significant hyperpolarizing shifts in the midpoint (*V*_1/2_) values with the WT-β1 (Figures 2A,B, Table 3). There were no significant differences in the apparent valence when comparing temperature (Table 3).

**Table 2:** **Open-state fast inactivation**.

	**τ −20 mV (ms)**	**τ −10 mV (ms)**	**τ 0 mV (ms)**	**τ 10 mV (ms)**	**τ 20 mV (ms)**	***n***
**22°C**						
WT-β1 Control	1.29 ± 0.11	0.72 ± 0.05	0.51 ± 0.04	0.38 ± 0.03	0.34 ± 0.02	8
WT-β1 Drug	1.26 ± 0.16	0.70 ± 0.07	0.46 ± 0.04	0.36 ± 0.04	0.28 ± 0.02	7
C121W-β1 Control	1.03 ± 0.13	0.70 ± 0.07	0.52 ± 0.04	0.42 ± 0.05	0.39 ± 0.03	7
C121W-β1 Drug	1.09 ± 0.10	0.72 ± 0.04	0.54 ± 0.03	0.47 ± 0.03	0.39 ± 0.02	5
**34°C**						
WT-β1 Control	0.61 ± 0.04	0.41 ± 0.03	0.29 ± 0.02	0.23 ± 0.02	0.20 ± 0.02	14
WT-β1 Drug	0.55 ± 0.05	0.37 ± 0.02	0.28 ± 0.02	0.23 ± 0.02	0.19 ± 0.01	11
C121W-β1 Control	0.89 ± 0.06	0.57 ± 0.04	0.42 ± 0.02	0.34 ± 0.02	0.29 ± 0.02	18
C121W-β1 Drug	0.89 ± 0.06	0.66 ± 0.05	0.51 ± 0.04	0.41 ± 0.04	0.35 ± 0.03	13

**Table 3:** **Steady-state fast inactivation parameters**.

	**Inactivation (20 ms)**			**Inactivation (500 ms)**		
	***V*_1/2_ (mV)**	***z* (slope)**	***n***	***V*_1/2_ (mV)**	***z* (slope)**	***n***
**22°C**						
WT-β1 Control	−55.7 ± 1.6	−2.7 ± 0.1	5	−68.7 ± 1.4^a^	−3.7 ± 0.2	10
WT-β1 100 Drug	−56.6 ± 1.6	−2.8 ± 0.1	6	−73.5 ± 1.3^a^	−3.5 ± 0.3	5
C121W-β1 Control	−56.0 ± 3.0	−2.4 ± 0.1	4	−66.1 ± 2.2	−3.8 ± 0.2	11
C121W-β1 Drug	−53.4 ± 4.9	−2.2 ± 0.1	4	−68.7 ± 1.7	−3.8 ± 0.2	7
**34°C**						
WT-β1 Control	−66.3 ± 0.3^b^	−8.8 ± 0.3	7	−72.8 ± 0.5^c^	−8.4 ± 0.5	7
WT-β1 Drug	−70.0 ± 0.4^b^	−10.3 ± 0.4	6	−76.5 ± 0.6^c^	−9.8 ± 0.6	6
C121W-β1 Control	−61.4 ± 0.5	−11.1 ± 0.4	7	−70.4 ± 0.6	−9.3 ± 0.5	7
C121W-β1 Drug	−63.6 ± 0.7	−12.1 ± 0.7	6	−70.3 ± 0.4	−9.7 ± 0.3	6

a P < 0.05 vs. WT-β1 Control at 22°C.

b P < 0.01 vs. WT-β1 Control at 34°C.

c P < 0.05 vs. WT-β1 Control at 34°C.

In contrast, there were no significant shifts observed in the *V*_1/2_ or apparent valence values of C121W-β1 with 100 μM lacosamide using 20 ms or 500 ms prepulse durations at 22°C or 34°C (Figures 2C,D and Table 3).

**Figure 3:**
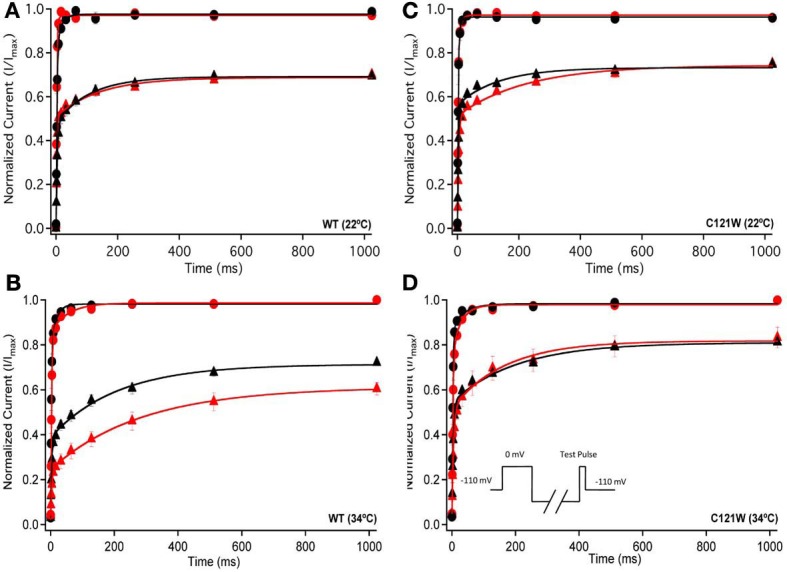
**Fast inactivation recovery**. Normalized Current is plotted against a range of recovery durations (ms). Filled circles shows fast inactivation recovery elicited by 20 ms prepulse durations; whereas closed triangles represent prepulse durations of 500 ms. For both WT at 22°C and 34°C **(A,B)** and C121W at 22°C and 34°C **(C,D)**. The means of control (shown in black) were compared to 100 μM lacosamide (shown in red). Panel **(D)** shows an inset of the double pulse protocol measuring fast inactivation recovery.

We examined the recovery kinetics of fast inactivation with both 20 ms and 500 ms prepulse durations. Peak current amplitudes in the second depolarizing pulse of a double-pulse protocol, plotted as a function of interpulse duration, were fit with a double exponential function. We compared the fast (τ_f_) and slow (τ_s_) time constants and amplitudes, and the asymptote (*y*_0_) values. There were no significant shifts in the kinetics of fast inactivation recovery at 22°C and 34°C with 20 ms prepulse durations (Figure 3, Table 4). With 500 ms prepulses at 34°C, there was a significant decrease in the amplitude of the fast recovery component with 100 μM lacosamide and also a significant decrease in the plateau *y*_0_ value (Table 4).

**Table 4:** **Fast inactivation recovery**.

	**Recovery (20 ms)**						**Recovery (500 ms)**					
	**τ1 (ms)**	**A1**	**τ2 (ms)**	**A2**	***y*_0_**	***n***	**τ1 (ms)**	**A1**	**τ2 (ms)**	**A2**	***y*_0_**	***n***
**22°C**												
WT-β1 Control	2.7 ± 0.3	0.82 ± 0.07	17.3 ± 9.5	0.15 ± 0.07	0.98 ± 0.01	5	3.7 ± 0.1^a^	0.48 ± 0.01	110.2 ± 10.1	0.21 ± 0.01	0.69 ± 0.01	5
WT-β1 Drug	1.6 ± 0.7	0.71 ± 0.07	3.6 ± 4.0	0.25 ± 0.07	0.97 ± 0.01	6	2.0 ± 0.1^a^	0.51 ± 0.02	140.5 ± 28.8	0.18 ± 0.01	0.69 ± 0.01	5
C121W-β1 Control	2.4 ± 0.4	0.83 ± 0.20	7.4 ± 7.9	0.12 ± 0.20	0.96 ± 0.01	4	3.3 ± 0.3	0.56 ± 0.02	133.7 ± 35.8	0.17 ± 0.02	0.73 ± 0.01	6
C121W-β1 Drug	1.9 ± 0.2	0.78 ± 0.09	8.5 ± 3.6	0.18 ± 0.09	0.97 ± 0.01	4	3.8 ± 0.3	0.52 ± 0.02	224.0 ± 53.5	0.23 ± 0.02	0.74 ± 0.02	5
**34°C**												
WT-β1 Control	1.8 ± 0.1	0.75 ± 0.04	17.0 ± 4.0	0.20 ± 0.04	0.98 ± 0.01	6	3.3 ± 0.3	0.36 ± 0.01^b^	207.0 ± 24.0	0.33 ± 0.01	0.71 ± 0.01^b^	7
WT-β1 Drug	2.1 ± 0.2	0.82 ± 0.03	23.0 ± 9.0	0.13 ± 0.03	0.99 ± 0.01	8	3.7 ± 0.3	0.20 ± 0.01^b^	285.0 ± 15.0	0.37 ± 0.01	0.61 ± 0.01^b^	6
C121W-β1 Control	2.5 ± 0.2	0.85 ± 0.03	40.0 ± 21.0	0.11 ± 0.03	0.98 ± 0.01	7	3.9 ± 0.4	0.51 ± 0.02	200.0 ± 44.0	0.26 ± 0.02	0.81 ± 0.02	7
C121W-β1 Drug	3.1 ± 0.3	0.73 ± 0.05	29.0 ± 9.0	0.21 ± 0.05	0.98 ± 0.01	8	4.2 ± 0.6	0.47 ± 0.03	184.0 ± 32.0	0.32 ± 0.03	0.82 ± 0.01	6

a P < 0.001 vs. WT-β1 100 μM Lacosamide at 22°C.

b P < 0.001 vs. WT-β1 100 μM Lacosamide at 34°C.

In contrast, we observed no statistically significant differences in any of the fast inactivation recovery kinetic parameter at either 22°C or 34°C in the C121W-β1 mutation (Figures 3C,D, Table 4) with both 20 ms and 500 ms prepulse durations.

### Slow inactivation

**Figure 4:**
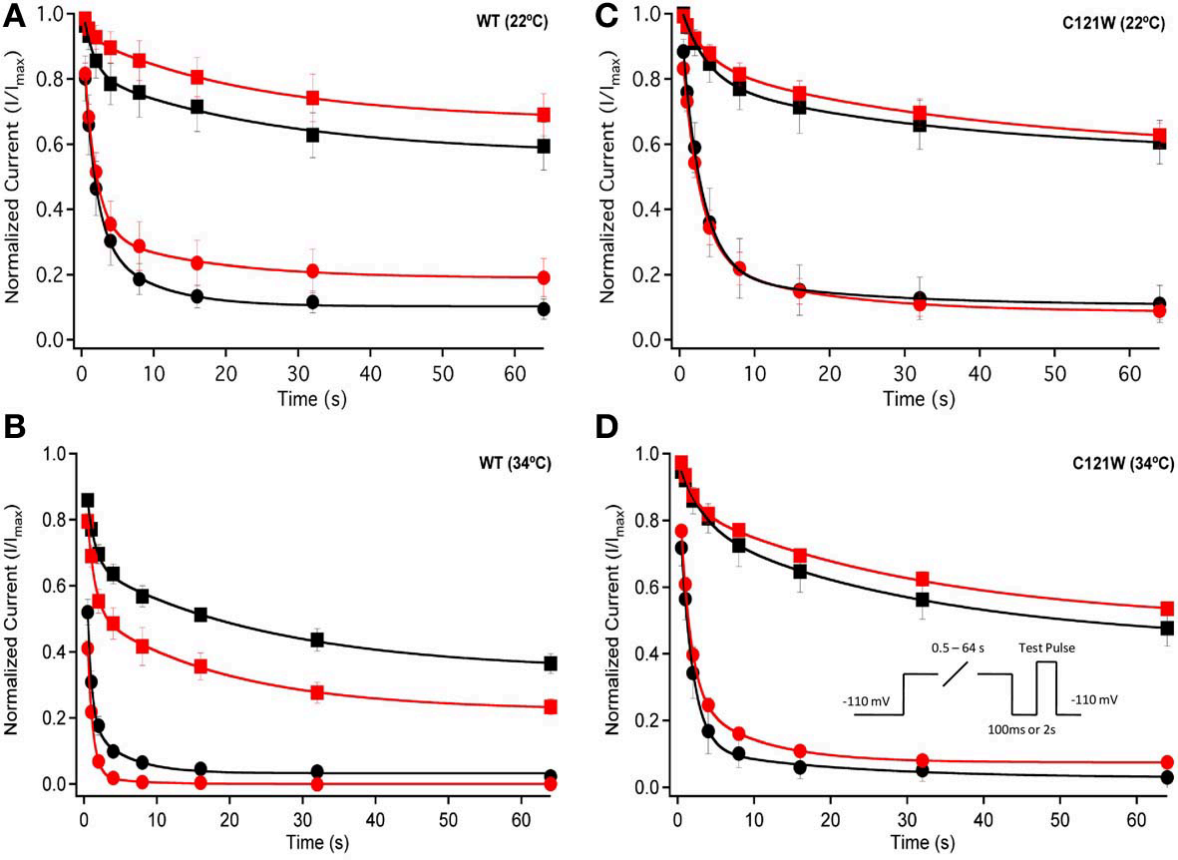
**Slow inactivation onset**. Normalized Current is plotted against a range of onset durations (s). We show two different recovery pulse durations: 100 ms (filled squares) and 2 s (filled circles) in control (black) and 100 μM lacosamide (red). For both WT at 22°C and 34°C **(A,B)** and C121W at 22°C and 34°C **(C,D)**. Inset in **(D)** shows the pulse protocol.

We examined onset kinetics of slow inactivation with either 100 ms or 2 s recovery pulse following conditioning depolarizations of varying durations. At 22°C the changes induced by 100 μM lacosamide were not statistically significant in either the C121W-β1 or WT-β1 co-expressed channels (Figures 4A,C, Table 5). Significant alterations of onset kinetics were observed at 34°C with the WT-β1 subunit. With the 2 s recovery pulse we observed an increase in the amplitude of the fast onset component as well as a decrease in the plateau *y*_0_ value (Figure 4B, Table 5). This trend was also observed for the 100 ms recovery pulse, but turned out to be not statistically significant. C121W-β1 co-expressed channels demonstrated no significant changes in the onset kinetics with drug perfusion at 34°C with either recovery pulse duration (Figure 4D, Table 5).

**Table 5:** **Slow inactivation onset**.

	**Onset (100 ms)**					**Onset (2 s)**					
	**τ1 (s)**	**A1**	**τ2 (s)**	**A2**	***y*_0_**	**τ1 (s)**	**A1**	**τ2 (s)**	**A2**	***y*_0_**	***n***
**22°C**											
WT-β1 Control	1.5 ± 0.3	0.47 ± 0.08	7.6 ± 2.3	0.23 ± 0.08	0.10 ± 0.01	1.48 ± 0.62	0.16 ± 0.03	25.8 ± 11.7	0.25 ± 0.03	0.57 ± 0.04	5
WT-β1 Drug	1.6 ± 0.1	0.48 ± 0.02	15.1 ± 4.2	0.14 ± 0.02	0.19 ± 0.07	1.03 ± 0.21	0.10 ± 0.01	23.9 ± 1.6	0.26 ± 0.01	0.67 ± 0.01	5
C121W-β1 Control	2.6 ± 0.2	0.68 ± 0.04	19.4 ± 13.7	0.10 ± 0.04	0.11 ± 0.01	3.35 ± 0.46	0.20 ± 0.02	33.7 ± 10.0	0.22 ± 0.01	0.57 ± 0.02	5
C121W-β1 Drug	2.3 ± 0.2	0.59 ± 0.04	16.2 ± 6.6	0.17 ± 0.04	0.09 ± 0.01	3.41 ± 0.60	0.14 ± 0.02	38.3 ± 9.8	0.27 ± 0.01	0.57 ± 0.03	7
**34°C**											
WT-β1 Control	0.59 ± 0.10	0.34 ± 0.04	4.2 ± 1.5	0.15 ± 0.04	0.03 ± 0.01	1.10 ± 0.20	0.19 ± 0.02^a^	25.0 ± 5.0	0.32 ± 0.02	0.34 ± 0.02^b^	8
WT-β1 Drug	0.73 ± 0.10	0.38 ± 0.01	5.4 ± 1.9	0.03 ± 0.01	0.00 ± 0.01	1.00 ± 0.20	0.28 ± 0.02^a^	19.0 ± 3.0	0.30 ± 0.02	0.22 ± 0.01^b^	6
C121W-β1 Control	1.60 ± 0.10	0.60 ± 0.03	19.0 ± 12.0	0.09 ± 0.02	0.03 ± 0.01	3.10 ± 0.80	0.16 ± 0.03	31.0 ± 8.0	0.36 ± 0.02	0.43 ± 0.03	6
C121W-β1 Drug	1.30 ± 0.10	0.50 ± 0.02	8.0 ± 2.0	0.20 ± 0.03	0.07 ± 0.01	1.70 ± 0.40	0.13 ± 0.02	31.0 ± 6.0	0.56 ± 0.02	0.49 ± 0.03	6

a P < 0.05 vs. WT-β1 100 μM Lacosamide at 34°C.

b P < 0.01 vs. WT-β1 100 μM Lacosamide at 34°C.

**Figure 5:**
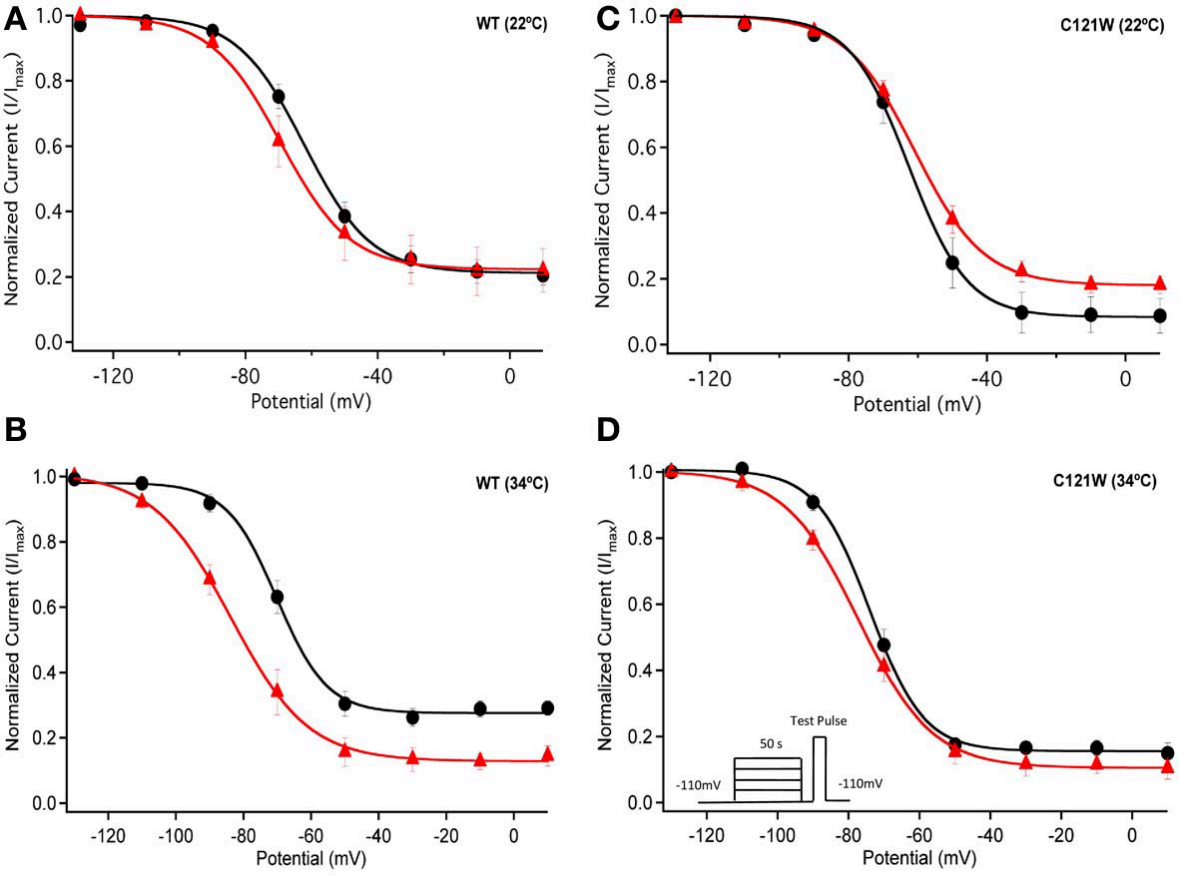
**Steady-state slow inactivation**. Normalized Current is plotted against the potential (mV). Control is shown in black circles and drug in red triangles. For both WT at 22°C and 34°C **(A,B)** and C121W at 22°C and 34°C **(C,D)**. Panel **(D)** shows an inset of the pulse protocol used to measure slow inactivation. The difference from the protocol used to measure steady-state fast inactivation is that the prepulse duration is longer (50 s) and followed by a brief recovery pulse that recovers channels from fast inactivation prior to the test pulse.

We next examined the voltage-dependence of steady-state slow inactivation. We observed significant effects of lacosamide at elevated and normal temperatures in the presence of the WT-β1 subunit. At 22°C there was a ~7 mV hyperpolarizing shift in steady-state slow inactivation in 100 μM lacosamide (Figure 5A, Table 6). This hyperpolarized shift was exacerbated at 34°C (Figure 5B, Table 6). The maximum probability of steady-state slow inactivation also increased significantly in the presence of the drug at 34°C (Table 6). C121W-β1 co-expressed channels exhibited no significant shifts in the *V*_1/2_, apparent valence, or the plateau values of steady-state slow inactivation at either 22°C or 34°C with the perfusion of 100 μM lacosamide (Figures 5C,D, Table 6).

**Table 6:** **Steady-state slow inactivation**.

	***V*_1/2_ (mV)**	***z* (slope)**	**plateau (*y*_0_)**	***n***
**22°C**				
WT-β1 Control	−61.9 ± 2.1^a^	−2.7 ± 0.4	0.21 ± 0.03	6
WT-β1 Drug	−68.9 ± 2.5^a^	−2.8 ± 0.4	0.22 ± 0.07	6
C121W-β1 Control	−62.3 ± 2.4	−3.4 ± 0.5	0.08 ± 0.05	5
C121W-β1 Drug	−60.7 ± 1.6	−2.7 ± 0.2	0.18 ± 0.03	8
**34°C**				
WT-β1 Control	−70.2 ± 0.9^b^	−7.4 ± 1.0	0.27 ± 0.03^b^	7
WT-β1 Drug	−83.8 ± 0.8^b^	−11.5 ± 0.7	0.12 ± 0.03^b^	9
C121W-β1 Control	−73.9 ± 0.5	−7.6 ± 0.5	0.15 ± 0.02	7
C121W-β1 Drug	−77.2 ± 0.5	−10.4 ± 0.4	0.10 ± 0.02	11

a P < 0.05 vs. WT-β1 Control at 22°C.

b P < 0.001 vs. WT-β1 Control at 34°C.

**Figure 6:**
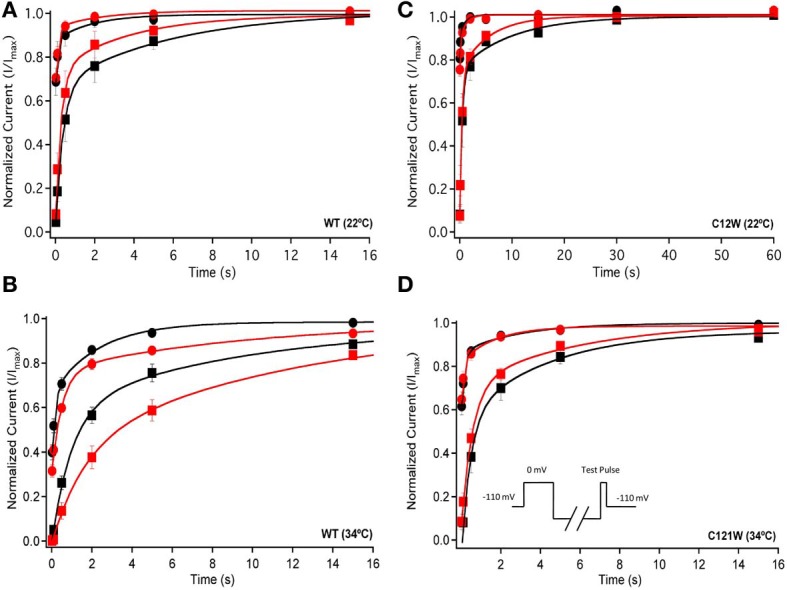
**Slow inactivation recovery**. Normalized Current is plotted over the various recovery durations (s). Recovery was measured using both 500 ms prepulse durations (closed circles) and also 8 s (closed squares). Control (black) is compared to 100 μM Lacosamide (red). For both WT at 22°C and 34°C **(A,B)** and C121W at 22°C and 34°C **(C,D)**. Panel **(D)** shows a pulse protocol inset used to measure slow inactivation recovery.

We also measured recovery from slow inactivation and drug block using both 500 ms and 8 s conditioning depolarizing pulses. At 22°C we did not observe any significant differences in any of the kinetic parameters of recovery with either 500 ms or 8 s depolarization durations with WT-β1 or C121W-β1 subunits with the addition 100 μM lacosamide (Figures 6A,C, Table 7). WT-β1 but not C121W-β1 co-expressed channels demonstrated a significant deceleration of recovery with drug perfusion at 34°C (Figures 6B,D, Table 7).

**Table 7:** **Slow Inactivation Recovery**.

	**Recovery (500 ms)**					**Recovery (8 s)**					
	**τ1 (s)**	**A1**	**τ2 (s)**	**A2**	***y*_0_**	**τ1 (s)**	**A1**	**τ2 (s)**	**A2**	***y*_0_**	***n***
**22°C**											
WT-1 Control	0.09 ± 0.02	0.19 ± 0.02	1.87 ± 0.68	0.12 ± 0.02	0.99 ± 0.01	0.38 ± 0.06	0.62 ± 0.05	5.89 ± 1.76	0.34 ± 0.05	1.01 ± 0.02	5
WT-1 Drug	0.13 ± 0.03	0.23 ± 0.03	2.31 ± 1.51	0.08 ± 0.03	1.01 ± 0.01	0.28 ± 0.07	0.64 ± 0.09	3.66 ± 1.95	0.26 ± 0.09	0.99 ± 0.02	5
C121W-1 Control	0.09 ± 0.06	0.12 ± 0.06	1.11 ± 1.19	0.09 ± 0.05	1.01 ± 0.01	0.49 ± 0.09	0.66 ± 0.06	9.98 ± 5.36	0.24 ± 0.05	1.00 ± 0.03	5
C121W-1 Drug	0.10 ± 0.06	0.13 ± 0.05	1.10 ± 0.73	0.12 ± 0.05	1.01 ± 0.01	0.41 ± 0.06	0.66 ± 0.05	5.58 ± 2.09	0.26 ± 0.05	1.00 ± 0.01	7
**34°C**											
WT-1 Control	0.16 ± 0.04^a^	0.27 ± 0.03^a^	2.3 ± 0.3^b^	0.32 ± 0.03^c^	0.98 ± 0.01	1.00 ± 0.20	0.58 ± 0.06	9.0 ± 2.0	0.38 ± 0.03^c^	0.96 ± 0.02	8
WT-1 Drug	0.51 ± 0.07^a^	0.44 ± 0.03^a^	10.0 ± 3.0^b^	0.22 ± 0.02^c^	0.98 ± 0.01	1.60 ± 0.40	0.42 ± 0.08	12.0 ± 2.0	0.57 ± 0.04^c^	0.98 ± 0.02	6
C121W-1 Control	0.16 ± 0.04	0.25 ± 0.02	2.9 ± 0.8	0.13 ± 0.02	1.00 ± 0.01	0.46 ± 0.09	0.60 ± 0.08	4.0 ± 1.0	0.41 ± 0.06	0.96 ± 0.02	6
C121W-1 Drug	0.12 ± 0.03	0.18 ± 0.02	1.8 ± 0.4	0.16 ± 0.02	0.98 ± 0.01	0.56 ± 0.08	0.62 ± 0.06	6.0 ± 2.0	0.29 ± 0.06	1.00 ± 0.01	7

a P < 0.001 vs. WT-β1 Control at 34°C.

b P < 0.01 vs. WT-β1 Control at 34°C.

c P < 0.05 vs. WT-β1 Control at 34°C.

## Discussion

In this study we established the role of sodium channel β1 subunit in modulating the effects of a physiologically relevant concentration of lacosamide on the biophysical properties of the brain sodium channel, Na_V_1.2. We also tested the functional effects C121W, a mutation in the β1 subunit which causes GEFS+ phenotype (Wallace et al., 1998), on the ability of lacosamide to inhibit sodium currents.

Similar to previous observations of Errington et al. (2008) and Sheets et al. (2008), we found that lacosamide caused no significant changes in the voltage dependence of Na_V_1.2 activation or kinetics of fast inactivation with either the WT-β1 or C121W-β1 subunits. Also there were no measurable differences in activation between the WT and the mutant C121W-β1 subunits. This result is consistent with findings of Qin et al. (2003) and Egri et al. (2012), who reported no effect on activation upon co-expression of the β1 subunit.

Lacosamide is postulated to be a slow inactivation-selective drug (Errington et al., 2008; Sheets et al., 2008) that does not affect fast inactivation mechanism but exerts its inhibitory effects on the time scale of seconds, far slower than that for many other “classic” anticonvulsants such as phenytoin, carbamazepine, and lamotrigine (Ragsdale et al., 1996; Ragsdale and Avoli, 1998). Thus, Errington et al. (2008) showed an insignificant shift in the steady-state fast inactivation curve with 100 μM lacosamide on N1E-115 neuroblastoma cells. Similarly, lacosamide had no significant effect on the steady-state fast inactivation profile in Na_V_1.7 or Na_V_1.3 (Sheets et al., 2008) expressed in HEK293 cells in the absence of β1 subunits.

The results of our study generally corroborate the notion of lacosamide's specific interaction with the slow inactivation. However, in the presence of the WT-β1 subunit we observed that even relatively short conditioning pulses (500 ms) are sufficient to cause a measurable effect of 100 μM lacosamide on the voltage dependent availability of the channels (Figure 2A). These findings are qualitatively similar to those of Uebachs et al. (2010) who described the importance of the β1 subunit in delimiting the sensitivity of Na_V_1.2 to another anticonvulsant, carbamazepine.

Co-expression of the WT-β1 subunit induces multiple effects on the kinetics of sodium channels, including accelerating fast inactivation and recovery, stabilizing steady-state fast inactivation (Tammaro et al., 2002; Aman et al., 2009; Webb et al., 2009; Egri et al., 2012), and increasing the maximum probability of slow inactivation (Vilin et al., 1999; Egri et al., 2012). Elevated temperature further accelerates the channel kinetics (Webb and Cannon, 2008; Thomas et al., 2009). As a result, at 34°C, channels enter inactivated states faster (Figure 2A vs. **B, C** vs. **D**) and, even with relatively short conditioning pulses, the effects of 100 μM lacosamide become statistically significant (Figure 2, Table 3). The trend seen in instantaneous availability curves is further supported by lacosamide slowing recovery after 500 ms conditioning depolarization at 34°C (Figure 3B).

Longer depolarization pulses favor accumulation of channels into the slow inactivated state and thus enhance the effects of 100 μM lacosamide (Figures 4–6). Also, the stabilization of steady-state slow inactivation by lacosamide is more pronounced at 34°C (Figure 5), suggesting that higher temperature also favors slow inactivation.

The C121W mutation is located in the Ig-like domain of the β1 subunit which is responsible for cell adhesion and aggregation, as well as α−β interactions resulting in modulation of the voltage dependence and kinetics in Na_V_ channels (Patino and Isom, 2010). A putative structural consequence of this mutation is the disruption of the disulfide bond between residues 21 and 121. Functional implications include destabilization of fast and slow inactivation leading to temperature-dependent hyper-excitability of Nav1.2/C121W-β1 channels (Egri et al., 2012). In the present study we demonstrate that the inhibitory effects of 100 μM lacosamide are lost when Na_V_1.2 is associated with the mutant C121W-β1 subunit at both room temperature and 34°C.

In conclusion, our results concur with the principal notion that lacosamide acts by stabilizing slow inactivation in Nav1.2. Our results go beyond previous studies to suggest the WT-β1 subunit indirectly modulates the efficacy of lacosamide by regulating the stability of the slow inactivation in Na_V_1.2 in a temperature dependent manner. The C121W-β1 mutation disrupts the thermoprotective role of the β1 subunit on channel availability, which leads to hyper-excitability observed in GEFS+ (Egri et al., 2012). This implies that patients carrying C121W-β1 mutation may not benefit from treatment with lacosamide.

### Conflict of interest statement

The authors declare that the research was conducted in the absence of any commercial or financial relationships that could be construed as a potential conflict of interest.
